# VATS Partial Pleurectomy Versus VATS Pleural Abrasion: Significant Reduction in Pneumothorax Recurrence Rates After Pleurectomy

**DOI:** 10.1007/s00268-018-4640-8

**Published:** 2018-05-01

**Authors:** Caecilia Ng, Herbert Thomas Maier, Florian Kocher, Silvia Jud, Paolo Lucciarini, Dietmar Öfner, Thomas Schmid, Florian Augustin

**Affiliations:** 1 Department of Visceral, Transplant and Thoracic Surgery, Center of Operative Medicine, Medical University of Innsbruck, Anichstrasse 35, 6020 Innsbruck, Austria; 2 Department of Hematology and Oncology, Medical University of Innsbruck, Anichstrasse 35, 6020 Innsbruck, Austria

## Abstract

**Introduction:**

Surgical treatment of primary spontaneous pneumothorax (PSP) usually consists of bullectomy and any form of pleurodesis to reduce risk of disease recurrence. Whether pleurectomy is superior to pleural abrasion is still a matter of debate with recurrence rates especially high when performed with a video-assisted thoracoscopic (VATS) approach. Aim of this study was to compare the efficacy of the two methods in prevention of recurrence of pneumothorax in a minimally invasive setting.

**Materials and methods:**

Between 01/2005 and 12/2015, 107 patients younger than 40 years with PSP underwent VATS bullectomy and either partial pleurectomy or pleural abrasion. Medical records of patients were reviewed retrospectively.

**Results:**

Pleural abrasion was performed in 34/107 patients, 73/107 patients underwent partial pleurectomy. There were no statistically significant differences in age, sex, body mass index or smoking history at time of surgery. There was no significant difference in major postoperative complications (*p* = 0.3022). Nine (8.4%) patients had a recurrence of pneumothorax during follow-up. Incidence of recurrence in those undergoing pleural abrasion was significantly higher than those undergoing apical pleurectomy (8/34 vs. 1/73, *p* < 0.001). Surgical technique was the only factor associated with a recurrence of PSP after surgical intervention.

**Discussion:**

In our analysis, a VATS partial pleurectomy proved to be effective for prevention of recurrent PSP. Recurrence rates were low despite a minimally invasive approach and significantly lower than in the pleural abrasion group. According to these findings, VATS pleurectomy might be considered as the primary choice for surgical pleurodesis in patients with PSP.

## Introduction

Primary spontaneous pneumothorax (PSP) mostly occurs in healthy individuals without an apparent cause, probably due to the rupture of subpleural emphysematous bullae located on the apex of the lung. Compared to PSP, a secondary spontaneous pneumothorax (SSP) occurs in the setting of underlying pulmonary disease, likewise COPD.

In the literature, open thoracotomy and pleurectomy remain the procedure with the lowest PSP recurrence rate [1]. Therefore, it is recommended in the currently available treatment guidelines of the British Thoracic Society [2]. Minimally invasive approaches tend to have higher recurrence rates. And yet, convinced by advantages like less surgical trauma, less pain, a shorter hospital stay and better quality of life in other thoracic diseases, an increasing number of surgeons choose a minimally invasive approach for treatment of PSP [3–7]. Whether apical pleurectomy or pleural abrasion performed via video-assisted thoracoscopic surgery (VATS) is superior for prevention of recurrence is still a matter of debate [1, 7–9].

Aim of this study was to compare the recurrence rate of pneumothorax after VATS bullectomy and pleurodesis either by partial pleurectomy or pleural abrasion.

In our department, we did perform pleural abrasion until 2009 for the prevention of recurrent pneumothorax. In 2009, the operative technique was changed to a minimally invasive partial pleurectomy. Primary endpoint of this study was the recurrence of pneumothorax after the first surgical pleurodesis.

## Materials and methods

### Study design

**Fig. 1:**
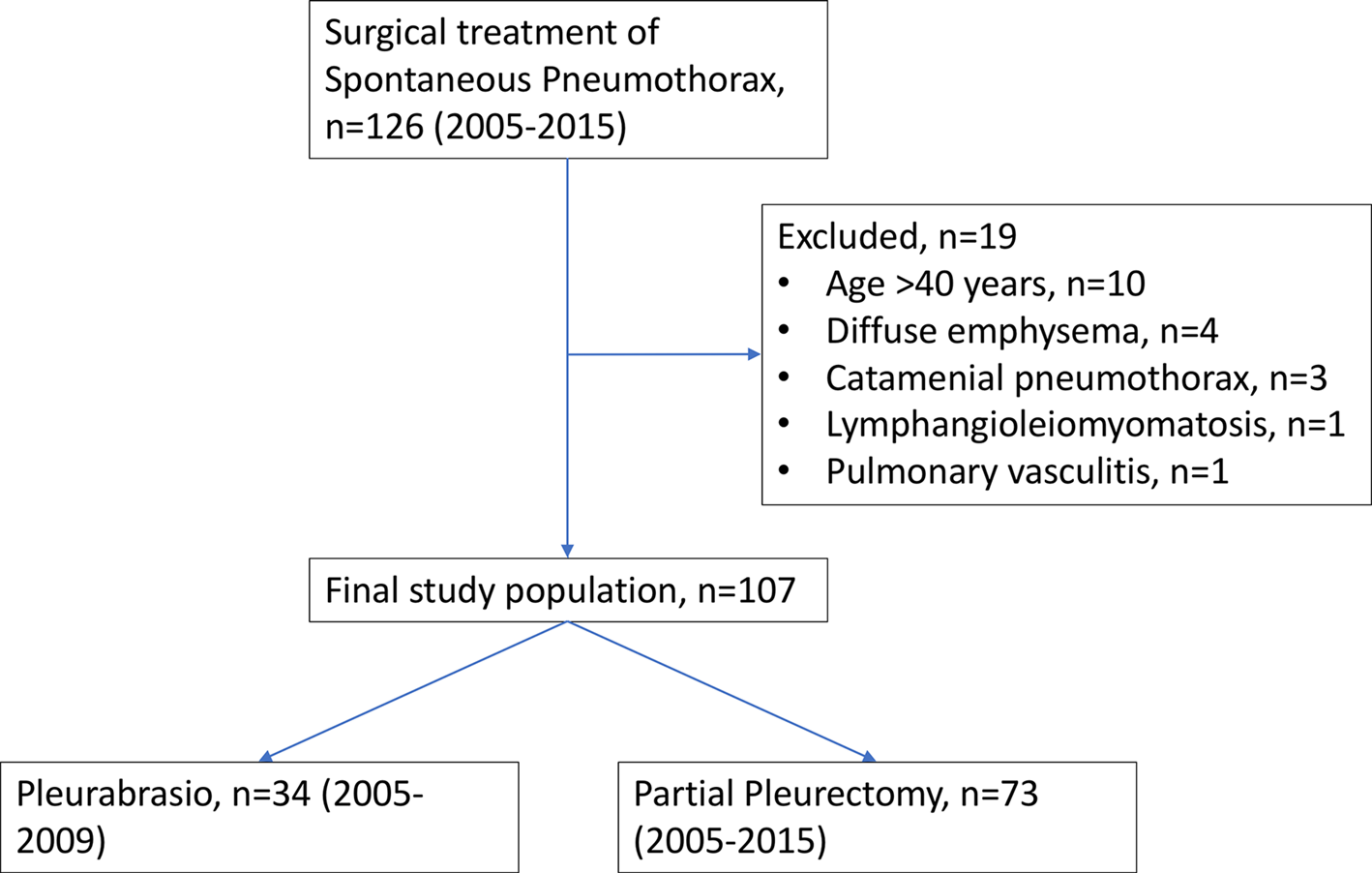
CONSORT diagram of all patients undergoing surgery for spontaneous pneumothorax and excluded patients due to the risk of underlying lung disease (secondary spontaneous pneumothorax)

Between January 2005 and December 2015, 126 patients underwent surgery for spontaneous pneumothorax (SP). Patients with secondary spontaneous pneumothorax (i.e., diffuse emphysema, catamenial pneumothorax, pulmonary lymphangioleiomyomatosis) or aged 40 years and older were excluded. A total of 107 patients with PSP were included for further analysis (Fig. 1). Indication for surgery was recurrence of SP (86 patients, 80.4%) or persistent air leak for more than 5 days (21 patients, 18.6%) during the first episode of PSP.

Patients’ characteristics including age, gender, body mass index (BMI), smoking habit, method of pleurodesis, chest drain duration, duration of hospitalization, postoperative complications, recurrence after surgery and follow-up were collected retrospectively. The study was approved by the local Institutional Review Board/Ethics Committee (AN2016-0155 364/4.18).

### Surgical procedures

Under separate lung ventilation, the patient is placed in a lateral position. We use a standardized anterior approach with three incisions for VATS as described previously [10]. The first skin incision for the camera trocar is placed in the 6th to 7th intercostal space at the anterior axillary line. Under visual control, the second incision is placed in the 4th to 5th intercostal space at the anterior edge of the latissimus dorsi muscle. The third 5 mm incision is placed in the 6th intercostal space and posterior axillary line. After inspection of the parenchymal surface and the pleural cavity, wedge resection of the bulla is performed using an endoscopic stapling device. Surgical pleurodesis is performed using one of the two methods: partial pleurectomy (PP) or pleural abrasion (PA).

**Fig. 2:**
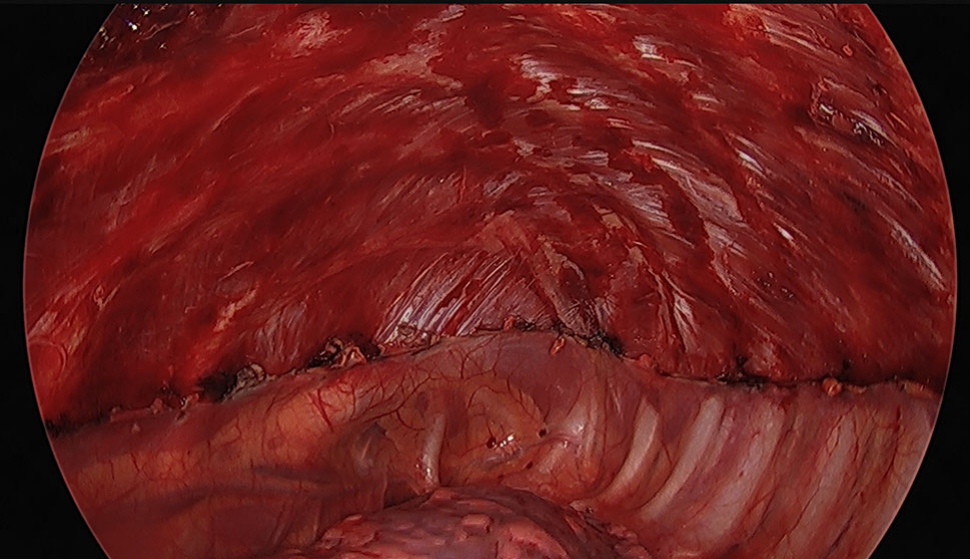
Intraoperative view after partial pleurectomy in the left thoracic cavity

PP is performed starting at the level of the anterior incision using blunt dissection. The dissection is carried out until the edge of intercostal muscles posterior and one centimeter lateral of the mammarian artery anterior to avoid damage to these structures. In the apex of the pleural cavity, the pleura is carefully dissected sparing the region of the subclavian artery and vein, again to avoid harm to these structures (Fig. 2).

PA is performed covering the same pleural area using a brush to cause petechial bleedings of the parietal pleura.

At the end of surgery one or two, chest tubes are inserted and connected to a water seal system. During the study period, we gradually decreased the number of chest drains. This was most likely an effect of other minimally invasive procedures where we considered only one chest drain to be sufficient. Overall, the number of chest drains was left at the discretion of the surgeon. Criteria for drain removal are no clinical signs of air leak and drainage of serous fluid less than 200 cc during 24 h.

Between 2005 and 2009, all but 3 patients did undergo PA. In 2009, we switched to PP after one of the faculty surgeons returned from a clinical fellowship. There were only 3 patients who had PA after 2009, and the rest was treated with PP. Two of 3 patients with PA after 2009 were performed by a senior surgeon and within a transition time of 6 months. The third patient with PA after 2009 had a suspicion of coagulopathy; therefore, no PP but PA was performed to reduce risk of bleeding. None of the patients had both procedures performed at the same time; it was either PA or PP.

Bullectomy and surgical pleurodesis are considered a teaching operation in our hospital. Therefore, a number of surgeons did operate on the patients in this study group. One of the board certified surgeons was present at all procedures either supervising or performing it.

### Statistical analysis

The qualitative parameters included the number and percentage of the various modalities. Data were expressed as mean ± standard deviation and median (range). Comparisons and the difference of recurrence rate between groups were performed using the Chi-square test or Fisher’s exact test, as applicable. All p values <0.05 were considered to indicate statistical significance. The statistics and analyses were evaluated by using SPSS software, version 20.0 (IBM Corp. Armonk, NY).

## Results

### Baseline characteristics

Between January 2005 and December 2015, 107 patients underwent VATS bullectomy and either PP or PA as a surgical treatment for PSP at our institution. Patients’ demographics were comparable between the groups and are shown in detail in Table 1.

**Table 1:** Patients’ demographics

Variables	PP (*n* = 73)	PA (*n* = 34)	*p* value
Sex			0.258
Male	53 (72.6%)	21 (61.8%)	
Age (SD)	24 (7.0)	24 (6.4)	0.151
Body mass index (SD)	19.7 (2.3)	20.3 (1.9)	0.503
Active smoking			0.877
Yes	33 (48.5%)	15 (31.3%)	
Number of PSP events prior to surgery			0.171
0	18 (24.7)	3 (8.8%)	
1	40 (54.8%)	20 (58.8%)	
2	12 (16.4%)	10 (29.4%)	
3 or more	3 (4.1%)	1 (2.9%)	
Follow-up duration (SD)	58.5 (31.4)	112.7 (41.6)	**<0.001**

Bold value indicate statistical significance (*p* < 0.05)

According to the number of PSP occurrences prior to surgical treatment, 60 (56.1%) patients experienced one, 22 (20.6%) patients two, 4 (3.7%) patients three or more episodes of recurrent spontaneous pneumothorax. Because of persisting air leaks, 21 (19.6%) patients were operated at the first event of PSP. There was no difference in the number of PSP occurrences between the two groups (Table 1). Due to the different time periods of treatment, follow-up periods of the two different groups differed significantly.

### Perioperative results

We found significantly more chest tubes placed at the end of the procedure in patients undergoing PA than PP (*p* = 0.014). Median drainage duration was 4 (2–7) days in the PP group and 3 (2–7) days in the PA group and did not differ between the two groups (*p* = 0.3437). Length of hospital stay was median 6 (3–11) versus 5 (2–13) days in the PP and PA group, respectively, with no significant difference between the two groups (*p* = 0.755). There was no in-hospital or 90-day mortality following any of the procedures.

Median follow-up period was 58.5 (SD 31.4) months in the PP and 112.7 (SD 41.6) months in the PA group and was statistically significant (*p* < 0.001), as expected according to the nature of the study design.

### Minor and major postoperative complications

During the postoperative course, a total of 6 (8.0%) patients of the PP group experienced complications. Two (2.7%) patients had minor complications including one patient with a Horner’s syndrome that resolved spontaneously within 2 months and one patient with signs of infection treated with antibiotics (Dindo–Clavien Grade II). Four patients (5.4%) with major complications required rethoracoscopy for hematothorax in three cases and bronchoscopy for atelectasis in one case (Dindo–Clavien Grade IIIB).

In the PA group, four patients (11.1%) experienced minor complications (signs of infection, Dindo–Clavien Grade II) that were all successfully treated with antibiotics.

With regard to major complications, there is no statistically significant difference between the two groups (4/73 vs. 0/34, *p* = 0.305). Perioperative data are summarized in Table 2.

**Table 2:** Perioperative characteristics

Variables	PP (*n* = 73)	PA (*n* = 34)	*p* value
Major complications	4 (5.47%)	0 (0%)	0.305
Number of chest tubes			**0.014**
1	57 (78.1%)	19 (55.9%)	
>1	16 (21.9%)	15 (44.1%)	
Postoperative chest tube duration (SD)	4 (1.3)	4 (1.5)	0.524
Length of hospital stay (SD)	6 (1.8)	5.5 (2.6)	0.755

Bold value indicate statistical significance (*p* < 0.05)

### Recurrence of pneumothorax after surgical pleurodesis

Nine (8.4%) patients had a recurrence of pneumothorax after surgical pleurodesis. Incidence of recurrence in those undergoing PA was significantly higher than those undergoing apical PP (8/34 vs. 1/73, *p* < 0.001). None of the other clinical variables, including the number of chest tubes placed at the end of surgery (1 vs. 2 chest drains, *p* = 0.279) did show any significant correlation with recurrence of pneumothorax after surgical pleurodesis.

Recurrence of pneumothorax occurred after a median of 9 (range 1–60) months in the PA group and after 2 months in the patient after PP. All patients were symptomatic with all but one experiencing thoracic pain and 6 out of 9 patients having dyspnea. All PA patients had a median of 4 (3.5–6)-cm apical pneumothorax, while the patient after PP did show apical adhesions (*p* = 0.002) on plain chest X-rays. Lateral distance between the lung and the thoracic wall was a median of 1.5 (0–5) cm in the PA group and 3 cm in the patient after PP (*p* = 0.593). Symptoms and size of recurrent pneumothorax after surgical pleurodesis are summarized in Table 3.

**Table 3:** Clinical symptoms and size of recurrent pneumothorax after surgical pleurodesis

Variable	PP (*n* = 1)	PA (*n* = 8)
Thoracic pain	1	7
Dyspnea	1	5
Pneumothorax size (cm)		
Apical	0	4 (3.5–6)
Lateral	3	1.5 (0–5)

No statistical test was performed due to the small sample size

## Discussion

According to the guidelines of the British Thoracic Society and the results of many other trials, an open approach via thoracotomy for surgical pleurodesis is considered the best surgical practice to reduce the risk of PSP recurrence. When compared to VATS procedures, thoracotomy is associated with lower recurrence of pneumothorax [1, 2]. However, due to less perioperative pain, shorter length of hospital stay and improved quality of life known from other indications, a VATS approach is preferred now by many surgeons [11]. At our institution, a minimally invasive approach is highly standardized and performed for different indications, including lobectomies, segmentectomies, pneumonectomies and even bronchoplasties [12]. Due to this standardization, it clearly became the preferred approach for many diseases, also for PSP. While the majorities of studies on surgical treatment of PSP were based on a comparison of thoracotomy and VATS, they did not primarily focus on different surgical pleurodesis procedures and their outcomes [5]. We performed a minimally invasive approach for surgical treatment of PSP since 2005 mainly using PA for surgical pleurodesis until 2009, when we switched to PP. With a median follow-up of 58 months for PP and 112 months for PA, we believe that this study population is suitable for a reasonable comparison regarding the success rate of surgical pleurodesis.

The study retrospectively analyzes 107 patients that are similar to patient cohorts from other published series including mainly male, thin and smoking young adults [13, 14].

During the study period, we offered surgical treatment to all patients who experienced more than one PSP episode, or in case of persistent air leak of more than 5 days. Regarding the indication for surgical treatment of PSP, we did use a rather conservative approach, not taking into account the recently reported 54% rate of recurrences or CT appearance of bullae during the first episode of PSP [15, 16]. However, this treatment algorithm should not influence postoperative recurrence rates or success rates of surgical treatment.

Possible postoperative complications might hinder some surgeons to adopt a PP: As suggested in the literature, PP comes with a slightly higher rate of postoperative complications, including bleedings and hematothorax with a need of reoperation [8]. We did find bleeding complications only in the PP group. However, there was no statistically significant difference in major complications between the two groups.

During the study period, we saw a significant decrease in the number of chest tubes placed at the end of the procedure. This might be most likely due to a larger experience in minimally invasive lung surgery, where we do usually not place more than one chest drain also for anatomic resections. According to our analysis, the number of chest drains placed at the end of surgery does not influence the recurrence of pneumothorax. Whether or not an additional chest drain would be sufficient to reduce the risk of hematothorax and the need for rethoracoscopy cannot be answered: Two of the patients needing reoperation had only one chest drain placed, and the third patient had two drains. To detect bleeding complications, we do rather focus on the quality of the drained fluid than the actual amount, which also explains a rather long chest drain duration and a longer length of hospital stay compared to other reports [9].

To further reduce the risk of bleeding, we now spare the area of the internal mammarian artery and the subclavian artery in the apex from PP (Fig. 1). We perform a meticulous control of hemostasis at the thoracic wall using a bipolar forceps to the point where no active bleeding is visible. Even though not statistically significant, we see a decreasing number of postoperative hematothorax within the last years as an effect of these considerations. The rate of major complications in our study is comparable to other published series [1, 13, 17].

In our study, we were able to clearly demonstrate a superiority of VATS PP compared to VATS PA. Moreover, all patients in the PA group who suffered a pneumothorax recurrence had no signs of apical adhesions on the chest X-ray indicating a primary non-responder to PA as surgical pleurodesis. No other clinical factor did show any correlation with recurrence of PSP after surgical pleurodesis.

To date, there are several studies reporting a favorable outcome with less recurrence after a VATS PP compared to PA; however, most of them failed to reach statistical significance. Ayed et al. reported in 2000 that 10.3% (4/39) of the patients of the pleural abrasion group suffered a relapse compared to 0% in the pleurectomy group [5]. Although these results did not reach a statistical significance, they show a tendency for less recurrences after pleurectomy (*p* = 0.05).

Chang et al. compared in 2006 the efficacy of PP to that of PA in the secondary prevention of PSP. While none of the patients in the pleurectomy group suffered from recurrence of PSP, 8.6% (3/35) in the PA group needed reintervention. Again, this study failed to reach statistical significance (*p* = 0.243) [17].

A study comparing PP to PA by Kocatürk et al. published in 2012 did show a significant reduction in pneumothorax recurrence after PP. However, 75% of the patients in this study were treated with thoracotomy. Other than the type of surgical access, results are comparable to our findings, indicating the reproducibility of superior results even with a minimally invasive procedure. Interestingly, Kocatürk et al. [18] also found a significant reduction in chest drain duration and hospital stay after PP compared to PA, which was not seen in our study.

In 2012, Bille et al. [1] published an article showing how different pleurodesis procedures affect the recurrence rate of PSP. They concluded that although the number of recurrences among the PA patients was higher, the difference to the PP group was not significant. Due to the limitations of the study, Bille et al. point out the need for further discussion on this subject.

In a systematic review of randomized controlled trials, Ling et al. [8] compared the effects of pleural abrasion with those of other interventions in the treatment of PSP in 2015. The authors concluded that pleural abrasion and pleurectomy result in the same recurrence rates, but pleurectomy shows greater postoperative pain and more frequent complications, such as postoperative acute bleeding. However, they also pointed out the limitations of their review, such as different follow-up and very variable recurrence rates in the control groups of the included studies.

Recently, Joharifard et al. [19] did show a significant reduction in recurrence of SP after pleurectomy compared to pleural abrasion in a pediatric patient population. Interestingly, this study did also show a rather high recurrence rate after pleural abrasion (40%). Reasons for the higher recurrence rate remain unclear, as no other clinical factor did differ between the two groups except for the surgical method of pleurodesis.

We do also find a rather high rate of SP recurrence after PA (23.5%). At this point, we can only speculate for reasons and would name efficacy of the treatment itself as shown in open surgical series [5, 13]. Inexperience and lack of standardization might also contribute, as the treatment of PSP is basically a teaching operation at our institution. However, as the incidence of recurrence in the PP group did not change over time and with experience, the low recurrence rate might be solely attributed to the technique itself.

In our study, we did not use a chemical (talcum) pleurodesis. Even though talc pleurodesis is widely used in Europe, it is not a routine procedure at our hospital. We do perform talc pleurodesis in patients with thoracic endometriosis and recurrent pneumothorax as well as other forms of secondary spontaneous pneumothorax. Due to possible side effects and complications in case of reoperation, we are rather reluctant to offer talc pleurodesis to young patients [2, 7]. However, in patients with coagulopathy we would recommend not to perform PP, but rather PA or talc pleurodesis to reduce postoperative bleeding risk.

### Limitations of our study

Retrospective data analysis is the main limitation of our study. Postoperative pain, shown to be higher after PP in other studies, was not evaluated in our cohort. Postoperative pain should, however, be considered in any prospective randomized trial in the future. The question remains, to what extend patients would prefer a higher pain score to an elevated risk of disease recurrence leading to the placement of a chest drain and reoperation.

In summary, we were able to show a significant reduction in pneumothorax recurrence after PP compared to PA. Even though PP was performed by a VATS approach, we achieved low recurrence rates comparable to PP with an open approach. Expertise gained from other minimally invasive thoracic procedures might have contributed to this success. Therefore, minimally invasive treatment of PSP should be performed in centers with high experience. This might improve patients’ outcomes twofold, reducing the risk of recurrence and also reducing the risk of postoperative complications. Evidence of superiority of PP is growing. And yet, large randomized trials should be conducted to support this hypothesis.
